# On the Effects of Temperature in Consumptive Disorders, in Answer to Dr. Sutton's Objections

**Published:** 1815-05

**Authors:** Isaac Buxton

**Affiliations:** New Broad-street


*For the Medical and Physical Journal.*

### *On the Effects of Temperature in Consumptive Disorders, in Answer to Dr. Sutton's Objections;* by Dr. Buxton.

I LATELY published some letters in the Monthly Magazine, the object of which was, to shew the ground that existed for the establishment of an Infirmary for Diseases of the Lungs. On these letters Dr. Sutton, in your Number for March, has made some remarks, to which I think it requisite to reply. The doctor commences by noticing the agreement which subsists between the Eclectic reviewer of Dr. Southey's late publication on Consumption and myself. Dr. Sutton appears to insinuate, though he does not openly express this opinion, that friendship had in some degree biassed the review. It certainly must be gratifying to my mind to observe a person of ability and knowledge supporting the same sentiments as I have maintained. The reviewer is evidently a man who thinks for himself. If similarity of opinions on most of the points under consideration will be sufficient to constitute a friendship between two individuals, then undoubtedly he is my friend. But further than than this, I do not know that he is so. In point of fact, I believe that I am totally unacquainted with this gentleman. Friendship, therefore, to myself, could have had no share in the guidance of his pen.

In my letter to the Monthly Magazine, the following are the points which I endeavoured to establish.

1. That asthma and consumption are very rare in hot climates.

2. That asthma is rare, but consumption not unfrequent, in mild climates.

3. That they are both very prevalent in this country, the climate of which is cold and variable.

4. That in this country they are much more frequent in winter than in summer.

5. That they have often been cured or relieved by the assistance of a high temperature (i. e. of about 65°) preserved in chambers during winter.

To support my first position, I have quoted a number of authors who have written on the diseases of tropical climates. Neither Chisholm, Hillary, Winterbottom, Curtis, Johnson, Mosely, nor Lind, speak of consumption or asthma as occurring in these latitudes. Lempriere gives tables of the deaths by different diseases in Jamaica, from which it appears, that one in twenty-seven died of consumption. He likewise mentions a single case of death from asthma.

Of the authorities to support the second position, Cleghorn and Townsend do not mention either of these disorders. Domeier speaks of them as occurring rarely. Neither Gourlay nor Irvine mention asthma; but both of them allude to consumption as not unfrequent.

Their frequency in England is proved by reference to different lists of diseases. Their fatality is shewn by the bills of mortality, confirmed by the registers of Gorsuch and Haygarth. The bills of mortality give between one-third and one-fourth of those who die, as destroyed by these complaints; the two gentlemen make the proportion to be between one-fourth and one-fifth.

Their greater frequency in winter than in summer was shewn by a reference to a register kept by myself, and to another given by Dr. Bateman in the Ed. Med. and Surg. Journal; by each of which it appears, that the cases of asthma and consumption are double in winter what they are in summer.

The benefit of a high temperature was evinced by the narration of nine unselected cases, where this remedy was used. Several of these individuals were cured of their complaints, plaints, and all, without exception, though some had been excessively ill, were considerably benefited.

I have thought it necessary to give this abstract of my arguments, to prove the utility of the practice in question, because the greater part of your readers will not have seen the original letters in the Monthly Magazine; but, until they have read this abstract, will judge of them through the medium only of Dr. Sutton's objections.

In the positions which I have endeavoured to establish, each article relates to asthma as well as to consumption; and the Infirmary for Diseases of the Lungs is open particularly to both these complaints. To the propriety of using a temperature of 65° during winter for asthma, Dr. Sutton makes no objection. I must consider that he was silent, because he had nothing to advance in opposition to this plan; and, therefore, that his silence is a tacit acknowledgment of the excellence of the remedy in question. Taking this, therefore, for granted, I shall say nothing more respecting asthma in the present letter.

With regard to consumption, Dr. Sutton has not attempted to controvert any of the positions above laid down; but has objected to some particular statements, and by arguments of a different nature has endeavoured to invalidate my conclusions. In answering the arguments of Dr. S. I find some difficulties, arising from different circumstances.

1. Although Dr. Sutton objects to my opinion of the causes why consumption occurs so frequently in England and in countries of a temperature nearly similar; yet he does not attempt to give his own explanation of this occurrence. From want, therefore, of accurately knowing his sentiments, I am in danger of misrepresenting them.

2. Although he has objected to the temperature which I have proposed, as in general most adapted to consumptive patients; yet he has not directly proposed in this paper any other which he considers more favourable. That he would employ a lower temperature than I have recommended, is evident, from the two cases he has related; but the exact degree, and whether it should be fixed or variable, he has left to conjecture.

3. Although the doctor proposes to follow a natural order, I find it rather difficult to discover that order; his remarks (excepting in the two first pages of the paper) appearing to be set down as they entered into the doctor's mind.

I shall endeavour to reduce what Dr. Sutton has said to separate heads, and shall answer each distinctly.

I. His first objection appears to me to be, that, although consumption occurs in England more frequently in winter than in summer, yet, as it is not nearly so prevalent in the northern cold climates as it is in England, that this frequency of occurrence depends not on the severity of the weather, but on its greater variableness during the cold than during the hot season. He particularly instances Denmark, Sweden, Russia, Holland, and Canada.

I have not leisure at this moment to examine the different authors who have written respecting the cold climates; but, as Dr. Southey (of whom Dr. Sutton speaks at the commencement of his remarks) has given a short statement relative to these countries, I shall refer to his work for an account of them.

Perhaps I may rather favour Dr. Sutton's argument by correcting his mistake with regard to the situation of Holland. It is not more to the northward than England. The accounts respecting the prevalence of consumption in that country are given differently by different authors. Dr. Cogan states the complaint to be very rare. Finke, on the contrary, mentions this disease as causing between one-fourth and one-fifth of the deaths at the Hague and at Bergen-op-Zoom. Where testimonies are so different, which are we to follow? Dr. Sutton chuses to follow Dr. Cogan alone. If it is really fact that Consumption is of rare occurrence in Holland, this must proceed from some peculiar cause, and must be considered as an exception to a general rule, the other countries both of Europe and America, which are placed in nearly the same temperature as England, being nearly or quite as liable to consumption as our island. With respect to Canada, Walsh does not advert to consumption, whilst Hearne speaks of it as being very frequent among the northern Indians. Dr. Sutton thinks it right to abide by the former of these writers, whilst he entirely neglects the latter.

In considering the effect of climate on diseases in tropical and in the milder countries, we have little else to do than to observe the changes of the atmosphere, and to note the diseases; because the inhabitants of those countries are much in the open air, and because their habitations are neither closed, nor warmed by fires. But, in the colder countries, other circumstances are to be taken in the account besides the temperature of the external air. The Russian climate is far more severe than ours. But it is to be recollected that the Russian rooms are preserved at a constant high temperature, so that, although the natives wear a large quantity of cloaths when exposed to the cold external air, when within doors they throw off nearly the whole. To this temperature, then, the Russian women and children are generally subjected jected throughout the winter; and even those men who go out to work during the day, still are preserved in this hot-house warmth during the night. Here, then, is much more than half the population of the country constantly in a summer equable temperature in the midst of winter. But, should any individual of those who are much exposed to the air be taken ill with consumption, the moment he is so disordered as to cease from work, that moment he will constantly breathe an atmosphere thus warmed. This application of heat to the healthy and to the diseased, will surely modify the diseases of the country.

Dr. Sutton acknowledges "that consumption commences and prevails most in the severe season of the year in this country." But, conceiving he had completely proved that severity, either alone, or conjoined with variableness, could not be concerned in producing consumption, he endeavours to attribute this greater frequency to variableness of climate alone during the "colder seasons, because the temperature then varies more than in the other parts of the year." Unfortunately, Dr. S.'s assertion is not consistent with fact. The temperature generally varies more in this country during summer than during winter. I examined the register of the weather kept by Cary for 1814, and recorded in Tilloch's Philosophical Magazine. In January, the difference between the highest and lowest points of the thermometer was 26°; in February, 30°; in July, 31°; in August, 26°. The average difference between the highest and lowest points in twenty-four hours was, in January, 5½°; in February, 10°; in July, 11°; in August, 12°. Hence, if the prevalence of consumption depended on the variableness of climate alone, that disease ought to prevail most in summer. But, as it actually occurs most in winter, I think I am justified in the conclusion that the severity then experienced, joined to variableness of temperature, may be considered as producing this difference. Supposing it could be proved that all persons exposed to the rigours of a Russian winter were less liable to consumption than if exposed to an English one, it might shew that consumption, like the plague, required for its greatest prevalence a certain temperature; and that the temperature of the Russian winter was too low, while that of an English winter favoured its production in the greatest degree.

II. Dr. Sutton's next objection appears to be, that consumption is of frequent occurrence in mild climates, and, therefore, that if we argue from the prevalence of this disorder in particular latitudes, a warm temperature cannot be

 beneficial beneficial in cases of this kind. In proof of this, he says, (p. 204) "Next to our own country, France and the south of Europe seem to be the regions most visited by this disease." This statement is very incorrect. Next to our own country, those parts of Europe and America whose temperature is nearly similar to that of England, are subject to this scourge. In proceeding towards the equator, the milder countries have the next place; and, lastly, those within the tropics, where this disease prevails least. Respecting the mild climates, Dr. S. dwells much on what is said by Irvine and Gourlay relative to Sicily and Madeira, these authors having spoken of consumption as of frequent occurrence in those countries, whilst he does not mention Cleghorn, who entirely omits consumption when describing the diseases of Minorca, or Domeier, who considers consumption as of rare occurrence in Malta. But, taking Irvine and Gourlay as the standards, whoever, with an unbiassed mind, reads their respective publications, will find consumption spoken of as if it occurred much more frequently than in the tropical countries, but not nearly so often as in our island. These authors not having given us comparative lists of diseases, we can only judge of the frequency and fatality of any complaint by the general manner in which it is spoken of. If a foreigner were describing the disorders of England, he surely would place consumption in a most prominent point of view. But neither Irvine nor Gourlay do so respecting this complaint. They both treat of several disorders before speaking of consumption; and Irvine describes the summer and autumn, the latter particularly, as the most fatal periods of the year in Sicily, because then the most destructive fevers abound. Each of these authors, likewise, describes the country of which he is treating, in distinct and unequivocal terms, as much more favourable to consumptive patients than England. Their testimony on this head Dr. S. passes over in silence.

In this part of his paper, Dr. S. endeavours to prove that my statement of the rarity of consumption in tropical countries is incorrect, by observing (p. 204), that, "respecting Jamaica, the most clear and authentic account on this subject contradicts the vague representations of others, and the doctor (Buxton) is pleased to find, as a solution of the cause of more consumption being found to prevail in that island, that it varies more in the degrees of temperature than any other in the West Indies.' He makes the like remark respecting Sicily." This is rather an easy way of cutting the knot. A Hillary, a Lind, a Johnson, &c. are vague and deficient ficient in clearness and authenticity; and, therefore, their testimony is to be disregarded! Dr. Sutton ought to give rather more proof of their deficiencies than his bare assertion, before he sets their testimony aside. But, resting the question on Lempriere's statement, we shall find that one in twenty-seven deaths in Jamaica is caused by consumption. Supposing this to be the ratio in tropical climates, I should, I think, be correct in my assertion, that consumption is there a rare disease. But, if this complaint occurs more frequently in Jamaica than in other West India islands, and if this is the reason why it is mentioned by Lempriere, who wrote on Jamaica, and not by other authors, who wrote respecting other islands, is my conjecture improbable that this frequency depends on the greater variableness of its temperature? Dr. S., at the conclusion of p. 203, asserts variableness of temperature to be a cause of consumption; and he has not attempted to deny that Jamaica and Sicily are more variable in their temperature than most other countries in their respective latitudes. Yet, while he cannot controvert the facts, he attempts by a sneer to get rid of the conclusion, or rather conjecture, grounded on these facts. If Dr. S. could shew the falsehood either of the facts or of the conjecture, he would do much more to establish his point.

III. Dr. S.'s next argument is in the form of a question (p. 205): "Do patients more frequently recover from this disease in any certain climate?" He then shews that invalids frequently die of this disease in Madeira and Sicily; a circumstance never controverted. Yet even here, in quoting Dr. Gourlay, he makes Dr. G. express himself more strongly than he actually did. In saying, "that whole families in this island are swept away by it," (i. e. consumption,) he forgets the qualifying words "*at times,*" employed by Dr. G. I hope, under another head, to shew that patients do recover more frequently from this complaint in certain climates, and to that head I refer the reader.

IV. Another argument brought forward by Dr. S. is, that, although cold and variable weather may operate, with other occasional causes, to produce consumption, this is not sufficient to shew that an opposite condition of temperature should be adopted in the treatment of the disease. I have no where asserted, that from the cause we may safely argue immediately to the treatment. As a general maxim, this mode of argument would be fallacious; yet it frequently holds good, and this especially in diseases which prevail chiefly in certain regions. Thus, hepatic affections are induced and supported by heat of climate, and frequently cannot be cured till the patient is removed to a colder coun-

 try.

try. Ague is continued in consequence of the presence of the miasmata, by which it was first generated, and frequently requires removal to a different place in order to its cure. And it appears by no means improbable, that the same kind of weather which generates consumption also tends to hinder its cure. Those, however, who read either the letters in the Monthly Magazine, or the present answer to Dr. Sutton, will easily perceive, that, although this may assist as a collateral argument, or as introductory to others, I rest the force of the controversy on direct and positive arguments.

Here it is necessary that I should rectify a mistake of Dr. Sutton's, where he supposes that I have advanced (p. 206) "a sort of refutation of an opinion that he had given. He refers to his intimation that a certain humidity of atmosphere is desirable to the consumptive, &c." I had no intention of refuting the doctor's opinion. The evidences which I possess on this subject are not sufficient to enable me as yet to form a decided judgment. In my letters, I endeavoured to collect a number of authorities, and thence I deduced some remarks, striving not to offer any remark besides what I conceived to be actually contained in my authorities. In the close of my first letter, speaking of the tropical countries, I said, "Whether the countries are moist or dry, marshy or the contrary, consumption and asthma appear to be equally rare." Although Dr. Sutton objects to this remark, he does not, with respect to regions within the tropics, attempt to contradict the assertion; I suppose because he was unable to do so. He speaks, however, of several examples in this island, which he conceives to be in point, but against some of which, I think, just objections might be adduced. However, as I am not here contending for a contrary opinion, I shall pass them by without remark. The Sirocco or south-east wind, which Dr. Sutton quotes as dry (p. 207), Irvine and Domeier speak of as being moist.

V. Dr. S. asserts, that the removal of patients to a warm climate, while labouring under consumption, has been attended with considerable detriment; and

VI. That the application of a cold temperature has been attended with benefit.

I place these arguments together, because they are so connected that it is scarcely possible to separate them. As these are points on which the main stress of the controversy must necessarily depend, it is requisite that they should be examined with some attention. On this head, Dr. S. brings forward a letter from Dr. Gladstone, a friend of his, who had served in the Mediterranean. It appears, from his account, that nine men, labouring under consumptive habits, were embarked embarked in England on-board his ship for the benefit of the Mediterranean climate, and others, having a tendency to these complaints, were retained on-board for the same reason. Unfortunately, Dr. G. has not mentioned the time when they arrived in the Mediterranean; but he speaks of these men as being exposed to the Sirocco, which wind, Dr. Domeier tells us, blows chiefly in September. It is, therefore, reasonable to suppose, that they arrived about the latter end of summer or beginning of autumn, perhaps nearly the worst time at which they could arrive, on account of the intense heat to which their exhausted frames would thus be exposed. Three of these nine died during one day's dry wind. (By this he means the Sirocco or south-east.) The other six were sent to Malta, to be invalided for a northern climate. Whoever has had an opportunity of seeing sailors who have been dismissed from a ship for consumptive complaints, must be fully aware that very few of these men long survive. As long as they are capable of work, they will be retained; but frequently a man will be able to do some duty long after consumption has commenced. I have seen many of these invalids both under my own care and under that of others, and have found that they almost universally died. That several of the men sent to the Mediterranean in Dr. Gladstone's ship, must have been in the last stage of the disease, is evident, from three of them having died in one day in consequence of the Sirocco. This wind is universally allowed to be excessively hot. No wonder that persons nearly worn out by disease should sink under its debilitating effects. At the end of almost any other complaint, where an equal degree of weakness had been induced, the same effect would follow from the same cause, and for a similar reason—the excessive exhaustion produced by the heat, independently of any pernicious effect which this wind might be supposed to have on the lungs. Whether the remaining six, who were sent to Malta, recovered or died, we are not told. It is not mentioned that any of the men who had a tendency to consumption, suffered in any respect from the climate. With the opinion which Dr. G. entertains, if they had become worse, they likewise would most probably have been sent to Malta. This may, perhaps, be considered as tending to shew that the climate agreed with them. The circumstances here adverted to, exactly accord with what I have observed in England. Persons in the last stage of consumption will be exhausted by sultry hot weather, in a very short space, while those who are in the incipient stage of the disorder generally suffer little, if any thing, at the same period. Dr. G. then mentions the case of an officer in consumption, consumption, who went to Constantinople during the winter, and there recovered. The winter is just the time when a warm climate is likely to prove beneficial. But surely this case does not oppose my opinions. Whilst at Constantinople, this gentleman would breathe in a temperature widely different from that of an English winter.

To shew the benefits of the application of cold, Dr. S. has selected two cases. Dean, the first of these, will not, I think, prove much in favour of the doctor's opinion. This man was very ill in December, 1813, and in the course of the month became worse; lingered in nearly the same state upwards of two months, i. e. to the end of February, 1814, and in another month, or at the end of March, was much improved in every respect. By adverting to a register of the weather, it will be found that it was severe from the latter end of December to the 20th of March, with the exception of a short period in February. On the 20th of March, the weather became warm, and continued so afterwards. This man appears to have followed the usual routine of what is frequently observed in complaints of the chest. During nearly three months, while the weather continued severe, "his disease appeared to vary in no considerable degree." But at the latter end of March, when the weather became warm, he "improved in every respect." If a low temperature was as beneficial as Dr. Sutton would have us believe it, this man ought to have profited somewhat in more than two months' application of this salutary agent. Yet his complaint did not vary. And if warmth of temperature were so very prejudicial, even though he had become better during the cold weather, it might have been expected, that the pernicious agent (warm temperature) would have produced a baneful effect on him. This, however, did not ensue. On the contrary, under its application, he "improved in every respect."

In the other case mentioned by Dr. Sutton, the medical gentleman to whom he refers, appears to have been ill for some time with pulmonic disease; but, at the time Dr. S. saw him, to have had a vomica burst in his lungs, or an attack, somewhat acute, on an organ previously much disordered. The account given by the doctor is not sufficiently detailed to enable me to judge which of these conjectures is most consistent with his actual state. In either case the disease would run through a certain course before the patient could become better, particularly if a vomica had burst. In this condition, he is stated to have exposed himself during the day to a strong fire in a warm room, while during the night he was in a chamber which neither had a fire nor a ray of the the sun throughout the twenty-four hours, and consequently is described as having been cold. Can it be wondered at, that exposing himself to such vicissitudes he should become worse? in despair, he took to his chamber, where he "gradually got better" under a temperature of about 45°. Here, at least, the temperature was nearly equable. But the patient's amelioration was not a recovery, it was only such as to bring him back to the state in which he was previously to this attack.

I shall now endeavour to prove, that warmth of temperature is generally beneficial, and coldness of temperature hurtful in the cases now under consideration.

In my letters to the Monthly Magazine I have given the unselected cases of nine patients labouring under pulmonic disease, who were kept in a chamber warmed to about 65° during a portion of the winter, and this with decided benefit. I could cite the instances of individuals, who, in incipient phthisis, received the most marked advantage from going during winter to a warm climate. I could likewise quote the cases of several persons in nearly similar circumstances, who evidently fluctuated with the change of weather in winter, being relieved by mild and injured by severe temperature. But, that I may not swell this article to an enormous length, I pass these by to notice two strong authorities, and the grand experiment which is made every year in this country by the alternate recurrence of summer and winter. Single cases, particularly if selected for an individual purpose, will often deceive us. But experiments made on a scale like this, if accurately tried, scarcely can fail.

The testimony of Dr. Gourlay and Dr. Irvine is very decided respecting the beneficial effects of a removal from the cold of England to those comparatively warm climates on which they wrote. The former asserts (p. 90) that Madeira is "highly beneficial in consumption with the natives of other countries," evidently referring chiefly to the natives of England, the country from which he came, and in which he published his book. Dr. Irvine, in speaking of Sicily, (p. 93,) says, "I have seen the progress of many cases of phthisis arrested, and many men recover, for a time at least, a perfect state of health, who would, in all probability, have died in England." And (p. 99) "The phthisical invalid will find the Sicilian climate, if not the best in the world, at least greatly more congenial to his frame than the fogs and rains of England." To the testimony of these men, I think Dr. Sutton cannot object, since he can place the greatest confidence in them on other occasions, where he conceives that they favour his opinions. And, it appears to me, that, let Dr. Gladstone's letter have what weight it may, their assertions will more than abundantly counterbalance it.

I shall now appeal to the changes produced in the consumptive during the alternations of summer and winter. Consumption is a disease which rarely destroys its victim in less than three months, and frequently it continues a much longer space of time. During January and February the number of patients labouring under consumptive complaints and registered in the lists of medical practitioners is considerable, much more considerable than in the hot months of July and August. If warm weather were hurtful in this complaint, it is to be expected that as the summer advanced those patients whose disorders commenced in the winter would become worse, and, probably, would be exhausted and die in the hot months of July and August; but we find, in point of fact, that the number of deaths by consumption in these months is very much smaller than in the cold months of January and February; while, at the same time, the converse of this is equally true, that, though the number of consumptive cases under medical treatment is smaller in July and August, the number of deaths is much the greatest in January and February. In short, as the warm weather commences and continues, the deaths by consumption diminish; as the cold weather commences and continues, the deaths by consumption increase. It may, perhaps, be said, that the greater number of deaths in the cold months may arise from the greater number of patients who are seized with consumption during the cold weather. But we have before observed, that this disorder generally exists many months before it destroys the patient, so that most of those who die in January and February were ill during a considerable portion of the preceding year. That a greater number of consumptions begin in winter than in summer, Dr. S. allows; and, to shew that the number of deaths is greater, I shall refer to the London weekly bills of mortality. No. 5-12 for 1814, including the eight weeks from the 11th of January to the 8th of March, give 947 deaths by consumption. No. 29-36, including the eight weeks from the 28th of June to the 23rd of August, give 585 deaths.

Dr. Sutton inquires, (p. 205,) "Do patients more frequently recover from this disease in any certain climate?" The weekly bills give a decided answer to this question. The consumptive cannot die in so much greater numbers in cold than in hot weather, from change of treatment, for they continue under the same care and attendance at one period as at the other. The greater number of deaths cannot arise from greater variableness of temperature in winter than in summer, summer, for it has already been shewn, that the variation is greatest in summer. It cannot proceed from the greater dryness of the atmosphere in winter than in summer, for the hygrometer generally indicates more moisture in the former than in the latter season. The fact that there are so many more deaths in the cold than in the hot weather must be acknowledged; and the only reason of the fact which can be given is the cold of one period, and the comparative warmth of the other. It is of no consequence, whether the patient removes to another country, and thus goes into a warm climate, or whether, while he remains in the same place, the change of season brings warmth with it. The effect is precisely the same.

From what has been advanced, I think we may conclude, that a temperature cold and variable, like that of an English winter, is the great cause of consumption in this country; and, when the disorder is once formed, materially contributes to its fatal termination; and that, on the contrary, an equable warm temperature hinders the formation of the disease, and, when it is formed, is a remedy of great power to oppose its progress.

*New Broad-street;* ISAAC BUXTON.

*7th April,* 1815.

